# A usability study on mobile EMG-guided wrist extension training in subacute stroke patients-MyoGuide

**DOI:** 10.1186/s12984-024-01334-9

**Published:** 2024-03-21

**Authors:** Hao-Ping Lin, Yang Xu, Xue Zhang, Daniel Woolley, Lina Zhao, Weidi Liang, Mengdi Huang, Hsiao-ju Cheng, Lixin Zhang, Nicole Wenderoth

**Affiliations:** 1 Singapore-ETH Centre, Future Health Technologies Programme, CREATE campus, 1 Create Way, CREATE Tower, #06-01, Singapore, 138602 Singapore; 2 Department of Rehabilitation, Shengjing Hospital of China Medical University, 16 Puhe Road, Shenyang, Liaoning 110134 China; 3 Department of Health Sciences and Technology, Neural Control of Movement Lab, ETH Zurich, Gloriastrasse 37/39 GLC G17.2, Zurich, 8092 Switzerland

**Keywords:** EMG, Stroke, Neurofeedback, Mobile

## Abstract

**Background:**

Effective stroke rehabilitation requires high-dose, repetitive-task training, especially during the early recovery phase. However, the usability of upper-limb rehabilitation technology in acute and subacute stroke survivors remains relatively unexplored. In this study, we introduce subacute stroke survivors to MyoGuide, a mobile training platform that employs surface electromyography (sEMG)-guided neurofeedback training that specifically targets wrist extension. Notably, the study emphasizes evaluating the platform’s usability within clinical contexts.

**Methods:**

Seven subacute post-stroke patients (1 female, mean age 53.7 years, mean time post-stroke 58.9 days, mean duration per training session 48.9 min) and three therapists (one for eligibility screening, two for conducting training) participated in the study. Participants underwent ten days of supervised one-on-one wrist extension training with MyoGuide, which encompassed calibration, stability assessment, and dynamic tasks. All training records including the Level of Difficulty (LoD) and Stability Assessment Scores were recorded within the application. Usability was assessed through the System Usability Scale (SUS) and participants’ willingness to continue home-based training was gauged through a self-developed survey post-training. Therapists also documented the daily performance of participants and the extent of support required.

**Results:**

The usability analysis yielded positive results, with a median SUS score of 82.5. Compared to the first session, participants significantly improved their performance at the final session as indicated by both the Stability Assessment Scores (*p* = 0.010, mean = 229.43, CI = [25.74–433.11]) and the LoD (*p* < 0.001; mean: 45.43, CI: [25.56–65.29]). The rate of progression differed based on the initial impairment levels of the patient. After training, participants expressed a keen interest in continuing home-based training. However, they also acknowledged challenges related to independently using the Myo armband and software.

**Conclusions:**

This study introduces the MyoGuide training platform and demonstrates its usability in a clinical setting for stroke rehabilitation, with the assistance of a therapist. The findings support the potential of MyoGuide for wrist extension training in patients across a wide range of impairment levels. However, certain usability challenges, such as donning/doffing the armband and navigating the application, need to be addressed to enable independent MyoGuide training requiring only minimal supervision by a therapist.

**Supplementary Information:**

The online version contains supplementary material available at 10.1186/s12984-024-01334-9.

## Background

Stroke is the third leading cause of disability worldwide [1]. At least 50% of stroke survivors suffer from upper limb impairments that limit engagement in activities of daily living (ADLs) and reduce quality of life [2, 3]. There is a consensus that effective post-stroke upper limb rehabilitation benefits from high-dose repetitive-task training [4, 5]. Nonetheless, ensuring the delivery of sufficient training doses remains challenging, especially during the acute to subacute phases [6–10]. Additionally, it’s essential to emphasize the significant impact of treatment timing on post-stroke motor recovery. Research has shown that the optimal rehabilitation period occurs within the first 60 to 90 days following a stroke [11]. Furthermore, clinical trials on human subjects have consistently revealed that individuals receiving early intervention exhibit significantly better motor recovery outcomes compared to those who received delayed intervention [12–14]. Building on this understanding, recent studies [15, 16] also highlighted the potential of rehabilitation technologies in alleviating the burden of intensive and repetitive upper limb exercises for therapists. These technologies, facilitating high-dose upper limb rehabilitation during the early stages of stroke, even when the upper limb is still significantly weakened and unable to generate overt movement, may serve as a beneficial supplement to conventional treatment methods.

To address the unique challenges posed by post-stroke rehabilitation, various sensor-based technological solutions have been explored, such as the Leap Motion [17], Kinect [18, 19], and Myo armband [18, 20, 21]. While demonstrating promise in gesture recognition [22] and hand therapy with serious games [23, 24], the Leap Motion generally demands precise hand positioning and controlled lighting conditions [23, 25]. It is important to emphasize that the former may present practical challenges, particularly for individuals with highly impaired upper limbs, as they might encounter difficulty holding their shoulder and elbow in the necessary position for an extended duration. Kinect-based programs have also shown promise in upper limb rehabilitation [26–28]. However, they also exhibit technical limitations, including the complexity of use and dependence on the therapist’s assistance [19], along with challenges associated with occlusion [29], and reduced reliability for small movement amplitudes [29].

In this context, surface electromyography (sEMG) emerges as a versatile and promising avenue for post-stroke rehabilitation, alleviating users from strict positioning constraints and lighting considerations. Specifically, the integration of sEMG with the Myo armband introduces a wireless and highly portable dimension to upper limb rehabilitation. Furthermore, sEMG possesses the unique ability to detect movement intention in cases of upper limb paresis [30, 31]. Such capability is vital for stroke patients with limited active movement. The effectiveness of sEMG biofeedback has been demonstrated in prior studies, encompassing gait training [32, 33] and upper limb exercises, with notable advantages such as mitigating co-contraction patterns [34], achieving enhanced functional recovery outcomes compared to conventional therapy [35, 36], and receiving positive usability feedback from end-users [37]. The potential use of the Myo armband with gamified applications for upper limb rehabilitation is highlighted by recent studies in multiple sclerosis and stroke patients for hand/wrist rehabilitation [21, 38]. Furthermore, it’s worth noting that although the Myo armband is no longer on the market, alternatives continue to be available.

The usability of the Myo armband coupled with serious games has also been tested on both healthy participants [20] and health professionals [38]. However, its utility as a user-friendly tool in clinical settings for both stroke patients and healthcare professionals has not been thoroughly investigated. Furthermore, given the heterogeneity in the design of serious games, the generalizability of usability across different game types may be limited. To effectively pinpoint and address usability challenges, a rigorous and iterative evaluation process is required [39–41]. Previous studies have shown that inclusive participation of both patients and therapists has played a pivotal role in shaping the development of wearable exoskeletons [42, 43] and interactive game-based virtual reality systems [44].

Acknowledging this gap, our present research seeks to offer insights into the usability of the Myo armband integrated with a training platform specifically designed for post-stroke rehabilitation. In this study, we introduce ‘MyoGuide,’ a mobile platform with a serious game harnessing the benefits of sEMG-guided biofeedback training. Additionally, it incorporates a calibration feature to address the diverse nature of impairments observed among stroke patients. Our focus is on wrist extension training, a pivotal aspect for both activities of daily living (ADLs) [45, 46] and hand grasping actions [47, 48]. Additionally, wrist extension ability has been highlighted as a potential indicator of upper limb functional recovery [49].

In this study, our primary objective was to integrate usability assessments into the initial stages of MyoGuide’s development, targeting both stroke survivors and therapists. From stroke survivors, we aimed to gain insights into the overall usability of the training platform. From therapists, our goal was to assess whether MyoGuide serves as a tool that can be used in clinical settings. The overarching aim is to validate MyoGuide’s suitability for integration into clinical settings, particularly addressing challenges related to intensive and repetitive upper limb exercises.

## Methods

The central goal of this study was to integrate usability evaluations in the initial stages of MyoGuide development. Through usability tests involving stroke participants and collecting written reports from therapists, our objective was to identify potential usability challenges they might face. In this section, we will introduce our training platform (MyoGuide) and provide an overview of the experimental protocol, along with the inclusion/exclusion criteria of the study.

### MyoGuide mobile training platform

**Fig. 1:**
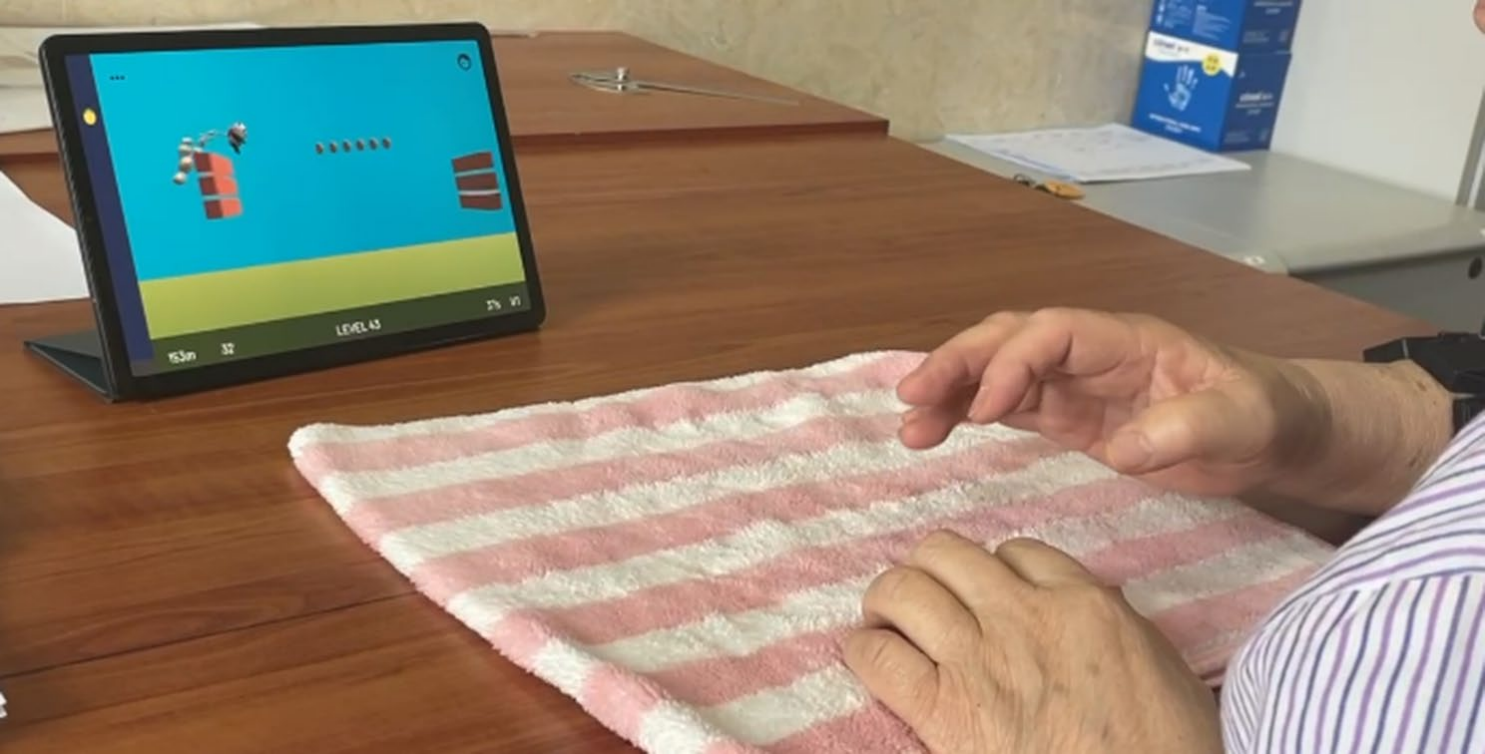
The gameplay of a participant. The video demo has also been submitted as supplementary material

The MyoGuide Mobile Training Platform consists of an EMG device for recording sEMG and a mobile device (tablet or phone) for visualising the recorded sEMG data in near real-time. The MyoGuide application which was used for data visualization was developed in-house (Unity Engine) and runs on the Android OS (Fig. 1). We used a Myo armband (Thalmic Labs) for recording sEMG. The Myo is a wearable 8-channel dry electrode BLE peripheral. Signed 8-bit raw sEMG data was transmitted from the Myo to a Samsung Galaxy Tab S5 tablet at a rate of 200 Hz.

Upon opening the MyoGuide application, the Myo armband was automatically paired with the tablet. A panel on the home screen of the application showed the pairing status, and the battery level of the Myo once paired. After a user profile was created by the therapist, the training tasks (stability assessment and dynamic task) were presented as a scrollable set of selectable panels on the right side of the screen. Other status information shown on the home screen included the user ID and session day. For this experiment the training tasks had to be completed in a specific order, so only the next training task to be completed could be selected. Panel colours were used to indicate the status of the task. A blue panel indicated that the task was selectable and the next task to be completed, a green panel indicated that the task had already been completed, and future tasks that could not yet be selected were displayed in grey. After selecting the training task to be completed, EMG calibration was performed in order to ensure that the EMG channel with the maximum range (max sEMG - min sEMG) was used to perform training.

sEMG from each channel was filtered in near real-time for signal visualisation. sEMG data was rectified, the mean of the 50 most recent samples was calculated, and the resulting value was Kalman filtered. The Kalman filter was updated at 60 Hz to match the refresh rate of the display, and the resulting value for each channel was used for visualisation. The filter pipeline was tuned to strike a balance between reducing noise while maintaining responsiveness.

### Experimental protocol

**Fig. 2:**
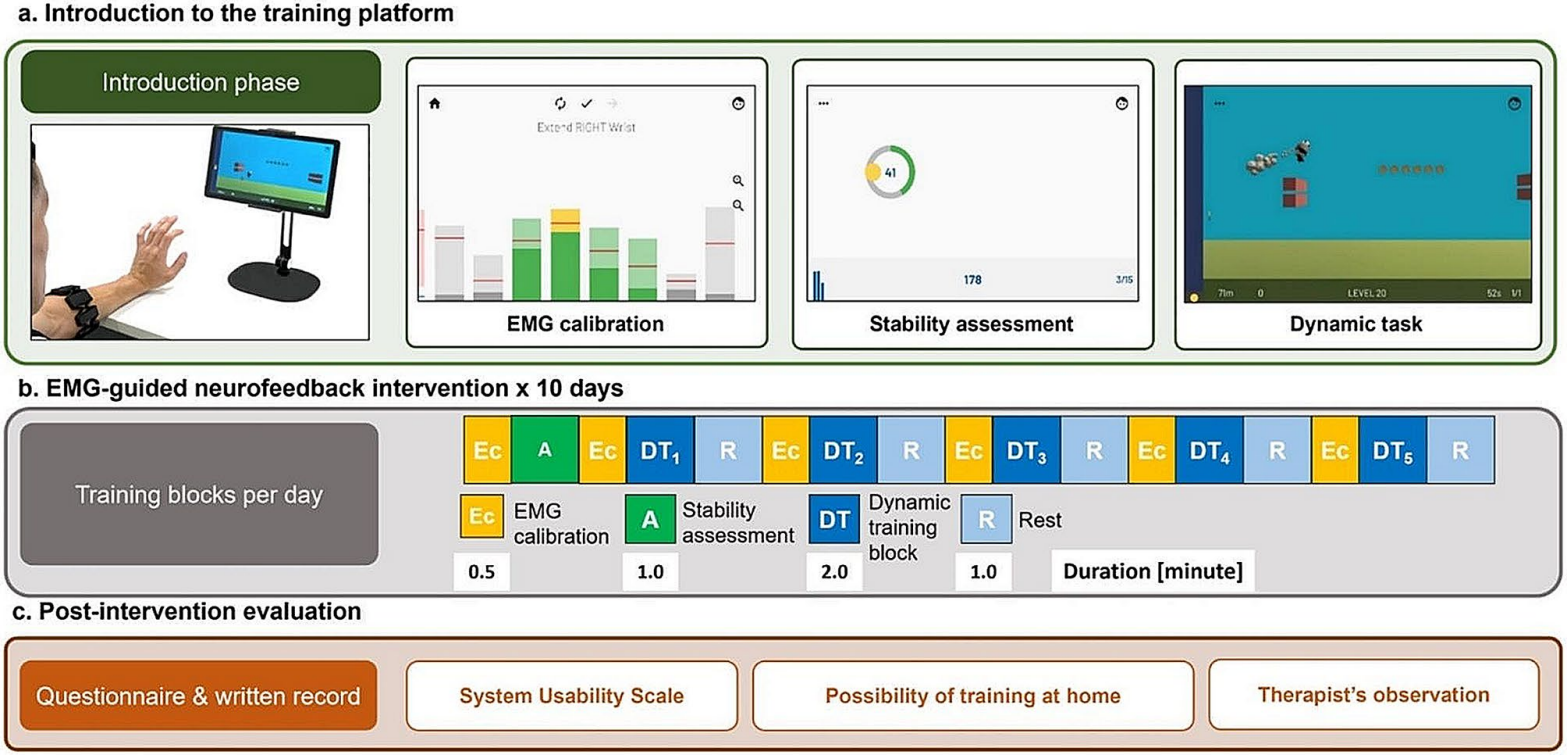
The study design and MyoGuide application. (**a**) Introduction phase: a therapist demonstrates the training program and EMG calibration provides reference values for controlling the yellow cursor in the stability assessment and the height and speed of the panda avatar in the dynamic task. (**b**) Intervention phase: participants underwent 10 days of training, consisting of EMG calibrations, stability assessments, and dynamic tasks (**c**) Post-intervention evaluation: the system usability scale and a survey on the possibility of training at home were administered to the patient. The therapist’s observations and feedback on training details were documented as well

The experiment protocol was conducted with a therapist-to-patient ratio of 1:1 in a supervised setting. The therapist assisted with MyoGuide and positioning the Myo armband on the stroke affected forearm. The Myo was positioned to capture sEMG signals originating from the forearm muscles, particularly the ECR muscle. The sEMG sensor featuring the LED icon was placed directly over the ECR muscle belly. The tablet was positioned in front of each participant on a table. Participants were seated in a comfortable manner, and arm support was provided when needed. The upper extremity neurofeedback training sessions, guided by sEMG signals, consisted of three distinct tasks: sEMG calibration, a stability assessment, and a dynamic task (as shown in Fig. 2a).

During the **calibration task**, participants were given instructions to perform a maximal voluntary contraction of the ECR muscle and hold the position for a minimum of one second. In Fig. 2a, the second subplot displays eight bars representing the sEMG channels. Among the middle four green-colored channels (which generally represent the electrodes placed over the extensors), the channel with the highest sEMG range (max sEMG - min sEMG) is identified as the “extensor channel” for performing the stability assessment and dynamic task.

After the calibration process a **stability assessment** was performed (depicted in the third subplot of Fig. 2a). The purpose of this assessment was to gauge the participants’ ability to regulate wrist extensor (ECR) sEMG activity using the extensor sEMG channel selected during calibration. To represent the participants’ sEMG activity level, a yellow cursor was employed. The position of the cursor was proportional to the filtered sEMG amplitude of the selected channel, where min sEMG was represented on the left side of the screen and max sEMG was represented on the right side of the screen. During the stability assessment, participants were tasked with controlling the cursor on the screen and positioning it within a target circle. They achieved this by actively controlling the sEMG level of their wrist extensors. The objective was to hold the cursor within the target area for a duration of three seconds. Each trial’s score depended on how long the cursor remained within the target area. We opted for a maximum score of 100 to reflect the percentage of time during which participants successfully maintained the cursor within the designated ring. We chose a percentage-based scoring system over a time-based one, as we believe it is more intuitive for gauging the participant’s success in keeping the cursor within the ring during the specified task duration. After completing a trial, participants were instructed to relax their wrist extensors and return the cursor to its starting position.

The stability assessment included three target areas, each associated with a specific calibrated maximum EMG level (25%, 50%, and 75%). The order of presentation for these target areas was randomized. Each assessment session comprised a total of 15 trials, with 5 repetitions allocated to three target areas. The participant’s proficiency in static sEMG control was evaluated based on the summed assessment score across all trials. The maximum attainable score was 1500 (i.e., 100 per trial for 15 trials), serving as an indicator of their overall performance and mastery of static sEMG control during the assessment.

In the **dynamic task**, presented in Fig. 2a, participants were assigned the objective of controlling a panda avatar by modulating their wrist extensor sEMG signals. The vertical and horizontal position of the panda avatar corresponded to the recorded sEMG level from the extensor channel, meaning that higher EMG signals led to the panda flying higher and faster. The participants were required to guide the panda towards rewarding coins placed at various heights while avoiding wall obstacles.

The task’s level of difficulty (LoD) spanned a range from 1 to 100, systematically increasing to challenge participants’ dynamic control over their extensor sEMG activity. To optimize the efficacy of the training tasks, we strategically adjusted various elements to modulate the LoD. These modifications included reducing the distance between obstacles and placing the rewarding coins in a less continuous fashion, prompting participants to engage in more frequent and rapid muscle contractions and relaxations. This adjustment aimed to increase repetition, a recognized crucial factor in stroke rehabilitation [4, 5]. Furthermore, we modulated the height of obstacles, requiring greater muscle activation to avoid collisions with the walls. This challenge was implemented to intensify the training, aligning with the principle that increased intensity contributes to more robust rehabilitation outcomes [4, 5]. The combined adjustments were designed to offer a progressive and tailored challenge, taking into consideration the dynamic needs and abilities of the participants undergoing the training.

Dynamic adaptability in difficulty was incorporated into the training regimen to enhance patient motivation and promote better recovery, as supported by promising findings in the literature [50–52]. This adaptive approach aimed to cater to the evolving skill levels and progress of individual participants. In our study, the LoD was dynamically adapted based on participants’ performance in the previous trial, considering factors such as the number of coins collected, and the distance traveled. A predefined threshold was set at 50% of the maximum achievable coins and distance, and if participants’ performance exceeded this threshold, the LoD was increased to provide a higher level of challenge. Conversely, if their performance fell below the threshold, the LoD was decreased to ensure an appropriate level of challenge. This adaptive approach allowed for personalized adjustments, aiming to maintain an optimal level of challenge and foster continuous improvement throughout training. The decision to set the threshold at 50% was informed by insights from our previous work, which explores the combination of muscle-computer-interface upper-limb training with bihemispheric transcranial Direct Current Stimulation (tDCS) [53].

The dynamic training sessions were structured in the following manner: Each training block, as shown in Fig. 2b, had a duration of 2 min, followed by a 30-second break. Participants completed five consecutive blocks on each of the 10 training days. On the final day of training, participants were asked to provide feedback on the usability of the system through questionnaires, allowing for an evaluation of their overall experience. During the training sessions, the therapist played a crucial role by closely observing the participants and documenting their observations, as depicted in Fig. 2c. This qualitative data collection provided valuable insights into participant engagement and overall experience during the training sessions. Each training session lasted approximately fifty minutes. Notably, the duration per training session would vary depending on the time spent on assessment tasks and breaks. The entire intervention was conducted over a span of two weeks, with training sessions scheduled for five days per week. This schedule resulted in a total of 10 sessions for each participant.

### Participants

To evaluate the usability of MyoGuide for early post-stroke wrist rehabilitation, we targeted subacute stroke survivors with functional deficits at the wrist of the affected arm. Given the nature of the Myo armband and MyoGuide training, we carefully designed the inclusion and exclusion criteria and would not include stroke survivors with elevated spasticity, as heightened spasticity could potentially hinder their ability to engage effectively in the training. To be included in the study, participants needed to meet certain criteria: (1) having experienced a mono-hemispheric, ischemic, or hemorrhagic stroke, (2) demonstrating measurable EMG activity in the m. extensor carpi radialis (ECR) of the affected arm, (3) having had a stroke between 2 weeks and 6 months prior to study inclusion, and (4) being older than 18 years of age. The exclusion criteria are: (1) having fully recovered wrist function (Fugl-Meyer Assessment of Upper Extremity wrist component (FMA-Wrist = 10), (2) experiencing enhanced spasticity (Modified Ashworth Scale (MAS) > 2), (3) having severe impaired vision or blindness, (4) exhibiting visuospatial neglect, (5) suffering from complete somatosensory loss, (6) having cognitive or communication impairment, or (7) having conditions that prevent informed consent or compliance. Our study participants were recruited at the Rehabilitation Centre at the Shengjing Hospital of China Medical University in Shenyang, Liaoning, China.

Ten participants were recruited in the study. However, due to technical issues during data transfer, we lost data from two participants. Additionally, one participant withdrew from the study due to a personal schedule change. Consequently, a total of seven participants (1 female, mean age 53.7 years, mean time post-stroke 58.9 days, mean duration per training session 48.9 min) successfully completed the training and provided comprehensive responses to the usability questionnaires. In addition to stroke survivors, three therapists were also involved in the study: one conducted eligibility screening, while the other two facilitated the training sessions and provided feedback on the system.

### Outcome measures

#### Primary outcome: system usability scale (stroke survivor’s perspective)

We specifically focused on evaluating the usability of our MyoGuide mobile training platform using the widely recognized System Usability Scale (SUS) [54]. The SUS questionnaire consists of ten items and is designed to assess overall usability, including effectiveness, efficiency, and satisfaction, of a system. We selected SUS for its attributes of being a quick, simple, reliable, and standardized tool for assessing usability [55, 56]. SUS has also been applied in assessing the technologies designed for post-stroke upper limb rehabilitation [57].

Participants rate their responses on a Likert scale ranging from 1 (strongly disagree) to 5 (strongly agree). To calculate the total SUS score, two separate calculations are performed. Firstly, the sum of the responses to the odd-numbered questions is subtracted by 5. Secondly, the sum of the responses to the even-numbered questions is subtracted from 25. These two results are then combined and multiplied by 2.5, resulting in the final total SUS score. The scores range from 0 to 100, with higher scores indicating better usability. A score between 51 and 71 is considered “OK”, a score between 72 and 85 is considered “Good”, a score between 86 and 91 is considered “Excellent”, and a score between 92 and 100 is considered “Best imaginable”. This evaluation was undertaken with seven subacute stroke survivors [58].

### Secondary outcome: possibility of training at home (stroke survivor’s perspective), training components and therapist’s feedback

In addition to the SUS questionnaire, we designed a supplementary survey to assess participants’ willingness to continue training at home, their overall attitude towards the system, and their confidence in independently using the device. This survey consisted of six items rated on a 5-point Likert scale, providing insights into participants’ perceptions and experiences with MyoGuide. The decision to create this supplementary survey was grounded in the objective of understanding participants’ willingness to continue using the training application, serving as an indication of potential future home use. Moreover, the survey included Myo-specific questions, such as ease of donning and doffing, to garner specific feedback on user-friendliness and practical aspects of the Myo armband.

Moreover, a comprehensive monitoring of various training components was conducted throughout the intervention period. The application employed in the study tracked participants’ stability assessment scores and the LoD during the dynamic task to effectively evaluate their aptitude for advancing within the training program.

Additionally, the therapist documented observations in a training survey, encompassing critical details such as the provision of assistance, the participant’s positioning during training sessions, and general observations. This feedback proved important in understanding the participants’ training experience, as well as providing further insights into their overall progress and level of engagement.

### Data analyses

To assess the temporal evolution of assessment scores and LoD, a linear-mixed effects model was employed using the lme4 package in R [59, 60]. This modeling approach also shown applicability even when dealing with relatively small sample sizes [61]. In our analysis, the session number was designated as a fixed effect, while the participant ID was incorporated as a random effect, accounting for individual variability. We conducted an analysis of the session effect by evaluating the impact of each time point. Firstly, we performed single-term deletions to identify any substantial differences among the different sessions. If we found statistical significance, we proceeded with post-hoc analyses utilizing Tukey’s test for multiple comparisons [62] between the baseline session with the remaining sessions to determine if there was any change in performance. Furthermore, to ensure the validity of our linear mixed-effect models, we conducted a residual analysis [63] to confirm that the assumptions of the model were not violated, including testing normality, and homoscedasticity of the residuals using the Q-Q plot and the Scale-Location plot, respectively. To further explore the relationships between different variables, we calculated Spearman’s correlation coefficients (rho). Specifically, we examined the correlation between SUS scores and the baseline characteristics and training performances of the participants. Furthermore, we investigated the correlation between baseline FMA-UE/FMA-Wrist scores [64] and the assessment score as well as between FMA-UE/FMA-Wrist and the changes in LoD during the dynamic tasks. For all statistics, a significance threshold of α = 0.05 was applied.

## Results

### Participants

Seven participants completed all ten training sessions and related questionnaires. Participants spent an average of 48.9 ± 13.1 (1SD) minutes per training session. Demographic data and baseline functional assessments of the upper limb are shown in Table 1.

**Table 1:** Participant demographics and baseline functional assessments

ID	Age	Sex	Days post-stroke	Affected hand	Stroke type	Stroke location	FMA-Wrist (max = 10)	FMA-UE (max = 66)
P1	65	M	39	L	I	Subcortical	0	8
P2	52	M	145	R	H	Subcortical	0	13
P3	52	M	46	R	I	Subcortical	4	28
P4	68	M	37	R	I	Subcortical	7	33
P5	52	M	42	L	H	Subcortical	7	35
P6	52	M	31	R	I	Subcortical	7	37
P7	35	F	72	L	I	Subcortical	7	53
**Mean**	**53.7**	**1 F**	**58.9**	**3 L**	**2 H**	**All subcortical**	**4.6**	**29.6**
**Median** **[Q1 - Q3]**	**52.0** **[52.0–58.5]**		**42.0** **[38.0–59.0]**				**7.0** **[2.0–7.0]**	**33.0** **[20.5–36.0]**

F = female; H = hemorrhagic; I = ischemic; L = left; M = male; R = right

### Usability analysis

#### System usability scale (SUS)

Participants generally offered favorable evaluations for the MyoGuide mobile training platform. The median SUS score in subacute stroke participants was “good” (82.5), as shown in Table 2. Among these participants, one individual assessed the platform as “excellent” (90), while six participants assessed it as “good” (72.5–85), and another participant as “OK” (70). Based on Q1, Q3 and Q9 from the SUS, participants generally conveyed their inclination to use the MyoGuide frequently and confidence in their ability to use the system. However, participants still expressed a desire for the availability of technical support during use in Q4. No significant correlations were found between the SUS score and age (Spearman’s rho = 0.012, *p* = 0.798), SUS score and FMA-UE score (Spearman’s rho = -0.070, *p* = 0.878), or SUS score and FMA_wrist_ score (Spearman’s rho = -0.05, *p* = 0.910). These results suggest that the perceived usability of the system was not influenced by age or baseline motor function.

**Table 2:** System usability scale for the myoGuide mobile training platform on subacute stroke survivors

	ID							
Survey Question	P1 (8)	P2 (13)	P3 (28)	P4 (33)	P5 (35)	P6 (37)	P7 (53)	Median
Q1: I think that I would like to use this system frequently.	5	4	5	4	5	5	5	4.5
Q2: I found the system unnecessarily complex.	1	2	2	2	2	2	2	2
Q3: I thought the system was easy to use.	5	4	5	2	5	5	5	5
Q4: I think that I would need the support of a technical person to be able to use this system.	5	5	4	4	5	5	4	4.5
Q5: I found the various functions in this system were well integrated.	5	4	3	4	5	5	5	4
Q6: I thought there was too much inconsistency in this system.	1	2	2	1	1	1	2	1
Q7: I would imagine that most people would learn to use this system very quickly.	5	4	5	4	5	4	5	4
Q8: I found the system very cumbersome to use.	1	1	1	1	1	1	1	1
Q9: I felt very confident using the system.	5	4	5	5	4	5	5	4.5
Q10: I needed to learn a lot of things before I could get going with this system.	1	2	1	2	2	1	4	1
**Total Score**	**90**	**70**	**82.5**	**72.5**	**82.5**	**85**	**80**	**82.5**

Likert scale: 1 (strongly disagree), 2 (disagree), 3 (neutral), 4 (agree), 5 (strongly agree). FMA-UE is given in parenthesis after the participant ID

### MyoGuide training

#### Level of difficulty (LoD) in the dynamic task

**Fig. 3:**
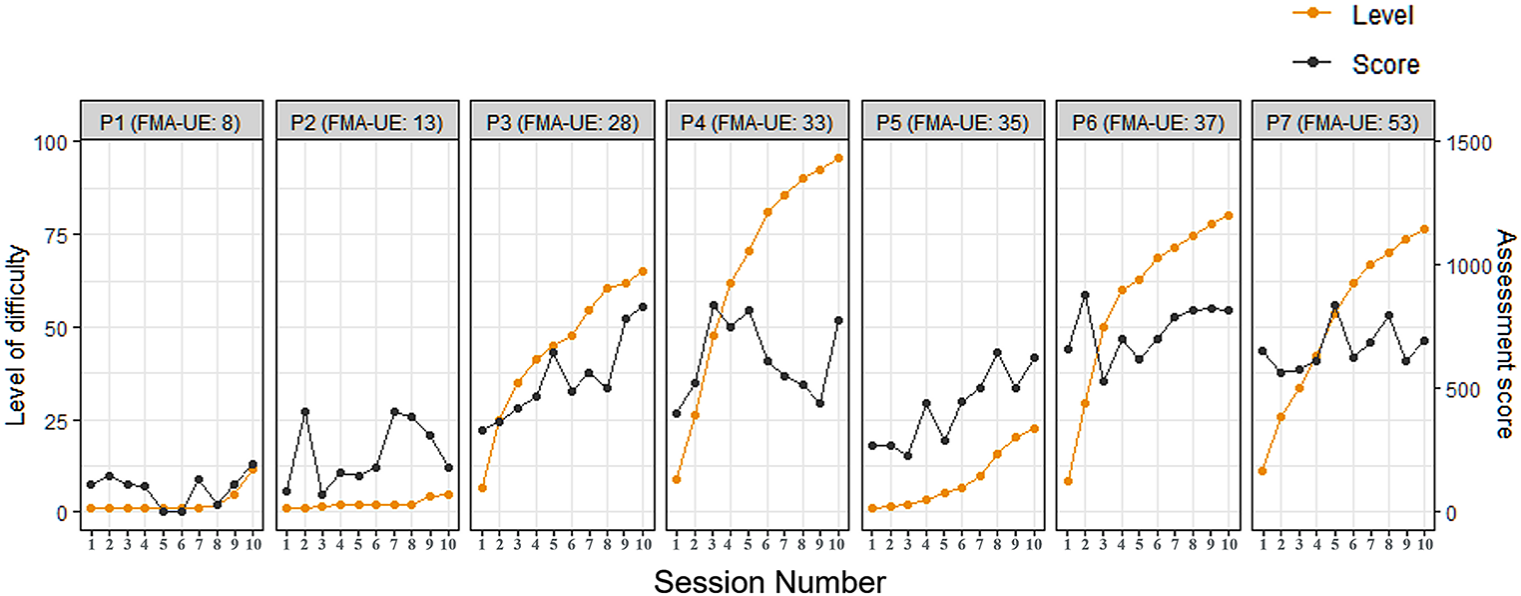
The progression of training parameters. The progression of both LoD in orange and assessment score in gray across the 10 training sessions2

**Fig. 4:**
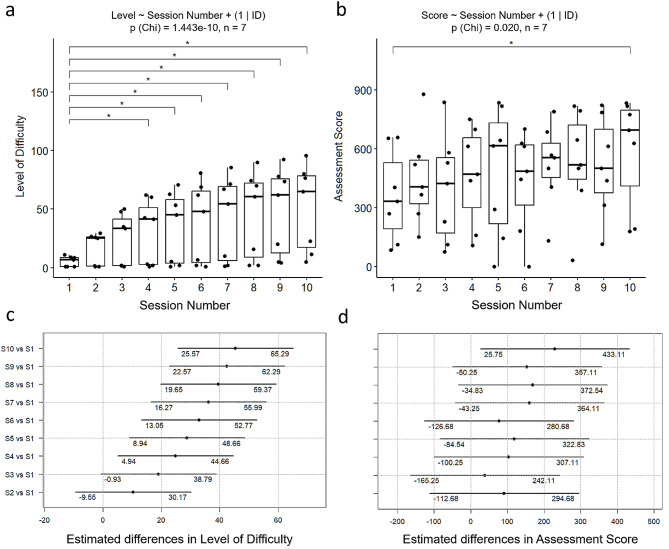
Comparison of sessions using single term deletion and Post-Hoc Tukey Test. Single term deletion shows significant differences from the baseline session starting from the fourth session in LoD (**a**). As for the Assessment Score (**b**), the presence of significant differences is only notable when comparing the final session with the baseline. Confidence intervals obtained through the Post-Hoc Tukey Test show the estimated differences between the baseline session and other sessions in LoD (**c**) and Assessment Score (**d**). The lower and upper bounds of the confidence intervals are presented below each corresponding line. Note that we treated subject IDs as random factors (1| ID), while session numbers were treated as fixed effects

During the 10-day training program, we observed an increase in LoD for nearly all participants, as shown in Fig. 3 (orange symbols). Utilizing a linear mixed-effects model, we found that the fixed factor “session number” reached statistical significance (*p* < 0.0001), indicating an increase in LoD across sessions. Further analysis using Tukey post-hoc tests revealed a significant difference in LoD starting from the fourth session (*p* = 0.003; mean: [24.80, CI: 4.94–44.66]) compared to the first session and continuing to the final session (*p* < 0.0001; mean: 45.43, CI: [25.56–65.29]) as shown in Fig. 4. These results indicate a substantial increase in LoD at the group level as training progressed. It is important to note that the increase in LoD was not consistent across all participants, with rates of progression being lowest for the two most impaired patients. This observation was further supported by a significant correlation between the changes in LoD and the baseline FMA-Wrist scores (Spearman’s rho = 0.800, *p* = 0.030) while the correlation between LoD and baseline FMA-UE scores of the participants did not reach significance (Spearman’s rho = 0.643, *p* = 0.120). This observation underscores the notion that individuals with varying degrees of impairment in the target region exhibited distinct progression in LoD. Furthermore, our analysis revealed no significant correlation between the change in LoD and SUS scores (Spearman’s rho = -0.020, *p* = 0.969). This indicates that better progression in LoD did not necessarily translate to higher usability scores.

### Assessment score in the stability assessment

Individually, we observed a positive correlation (Spearman’s rho = 0.857, *p* = 0.014) between participants’ baseline FMA-UE scores and FMA-Wrist (Spearman’s rho = 0.800, *p* = 0.030) and their average assessment scores, confirming. that individuals with less upper-limb impairment generally achieved better performance in the stability assessment task. No significant correlation was found between the mean assessment score and SUS score (Spearman’s rho = 0.160, *p* = 0.798), indicating that better performance in the stability assessment did not necessarily result in higher usability scores.

### Therapists’ observation and feedback

During the study, two participants (P1 and P2) performed training in a supine position, allowing for wrist extension without the influence of gravity, while the remaining participants conducted training in a seated position, performing wrist extension against gravity. The absence of any observed adverse events related to the training suggests the safety of the training protocol and the use of the MyoGuide system for subacute stroke patients. Specific narrative feedback from the two therapists (note that they are also co-authors) regarding the training platform is detailed below:

**Therapist 1** expressed that MyoGuide’s portability and ease of use distinguished it from traditional rehabilitation devices, making a strong impression. The therapist emphasized the potential for future applications in home rehabilitation and remote medical care. Notably, patients, particularly those accustomed to mobile devices, showed keen interest, quickly grasped training techniques, and exhibited greater initiative and confidence in independently operating the system. The combination of precise feedback and gamified tasks effectively motivated patients to engage proactively in training.

**Therapist 2** highlighted the excellent online feedback provided by the training setup, allowing patients to visualize their performance. The gamified environment for completing training tasks proved highly encouraging for patients. The setup’s simplicity and ease of understanding, coupled with its compact and lightweight design, enabled even early bedridden patients to participate. Despite potential fatigue during the game, the system’s daily difficulty adjustments facilitated most patients in persevering without discomfort.

### Future possibility of training at home

Furthermore, participants expressed interest in continuing the training at home. However, the survey also revealed challenges related to the use of the Myo armband and MyoGuide in an unsupervised setting. Specifically, participants mentioned difficulties in independently donning the Myo armband and operating the tablet. This indicates that using the MyoGuide platform without supervision will require adequate onboarding, initial guidance and potentially some support. Despite the participants’ motivation and positive experiences with training, practical aspects of using the technology independently require further development for its effective implementation in a minimally supervised environment.

**Table 3:** The possibility of training at home with technology

	ID							
Survey Question	P1	P2	P3	P4	P5	P6	P7	Median
I think the training improved my functional recovery.	5	4	5	4	5	5	5	5
I need others to help with putting on Myo.	5	5	5	5	5	5	1	5
I would like to continue the training.	5	4	5	5	5	5	5	5
I need others to help with operating the tablet.	5	5	2	5	5	5	1	5
I could perform the training independently.	4	1	4	2	3	3	5	3
I would like to take the training device home (if it is free of charge).	5	3	5	5	5	5	5	5

Likert scale: 1 (strongly disagree), 2 (disagree), 3 (neutral), 4 (agree), 5 (strongly agree)

## Discussion

The primary objective of this study was to incorporate usability evaluations into the early phases of MyoGuide development, utilizing a therapist-to-patient ratio of 1:1 in a supervised setting. By gathering insights through usability tests from stroke participants and written reports from the therapist, our aim was to uncover potential usability obstacles they might encounter. This approach not only facilitates the iterative enhancement of the training platform but also strengthens its utility as a clinical tool for therapists.

The usability report revealed the successful use of the MyoGuide training platform by all participants, including those with severe upper limb impairment (P1 and P2, with an FMA-wrist score of 0). Notably it also enabled training in various positions (supine or sitting). Stroke participants assigned favorable SUS scores ranging between good and excellent (70–90). The mean SUS score obtained in our study (80.4) compared favorably with similar studies exploring the usability of other novel rehabilitation technologies. For instance, the mean scores were 69.0 for arm and hand devices with a gaming environment [65], 70.1 for wearable soft-robotic gloves for stroke rehabilitation [66], and 71.9 for a robotic assistive device for home rehabilitation [57]. However, it’s important to acknowledge that SUS data were collected at the study’s conclusion to capture participants’ overall impressions. This timing choice may introduce bias to our results as participants became more familiar with the system over the study duration. Additionally, the supervising therapist assisted participants in navigating the application, potentially influencing their independent interaction with the training platform and introducing bias to the SUS results.

Early post-stroke rehabilitation is hypothesized to play a crucial role in promoting functional recovery and independence in ADLs [12–15]. However, active training is not easy to implement in clinical settings when patients are still severely impaired. Our findings indicate that the MyoGuide training platform shows promise for use in the early phase of stroke rehabilitation, particularly when a patient’s active movement of the wrist may be limited and might still be bedridden. The results showed that most of the patients could begin training with the platform under the guidance of a therapist following a short familiarization session, emphasizing its feasibility and potential advantages in supporting early-stage stroke rehabilitation.

In addition, all participants indicated that they would be willing to continue to train with the device at home. However, most participants expressed a need for assistance in navigating through the application and placing the armband, which posed a potential barrier to using the system in an unsupervised setting. While stroke participants acknowledged the benefits of the training, the practical aspect of using the technology without external support presented a challenge. These findings indicate that home-based rehabilitation systems will require adequate training of the patients and their caregivers, and that a remote support system might be essential.

Our study also examined the progression of two training components that can be easily assessed online: the assessment score in our stability assessment and the LoD in our dynamic task. LoD in our dynamic task increased with training and the post-hoc multiple comparison analysis revealed a significant increase in LoD starting from the 4th session compared to the 1st session. This signifies an overall adaptation to the training program, as participants were able to handle and perform tasks at higher LoDs as they progressed through the sessions. Notably, participants with moderate to mild impairment (FMA-UE: 31–66, based on [67, 68]) exhibited better progress compared to those with severe impairment (FMA-UE: 0–30), likely attributed to factors such as fatigue resulting from the frequent recalibration process. The impact of fatigue was evident as two participants with severe impairment (P1 and P2) could not complete all training blocks. Similarly, regarding the stability assessment, the correlation analysis demonstrated a significant positive correlation between the average assessment score and the baseline standard clinical assessment for the upper limb (FMA-UE & FMA-Wrist). Moreover, the assessment score did significantly improve across sessions with the assessment score in the 10th session being significantly higher than in the 1st session, indicating that participants did improve on the stability assessment over the course of training. However, it is crucial to note that the lack of a more rigorous randomized controlled trial (RCT) study design limits the ability to directly attribute these improvements to the training platform. A more robust RCT study design is necessary to further test the effectiveness of the training platform on motor recovery. To address the challenge of fatigue in the future, it is crucial to consider the individual abilities and limitations of patients with severe impairment during the calibration process. Additionally, considering that the placement of the armband remained unchanged throughout a session, the need for calibration before every task may not have been necessary. Future adjustments could involve skipping the calibration step, allowing users to continue with the initial calibration values. This would optimize the training process by minimizing unnecessary fatigue and providing a more efficient and personalized experience for patients with severe impairment. Additionally, implementing a more tailored approach to LoD, stratified based on the participant’s impairment level, could enhance the training experience. Participants with better function might begin with more challenging tasks, while those with higher impairment might require adjusted parameters to ensure meaningful participation, such as fewer training blocks, longer rest durations, and shorter task durations.

Through involving end-users early in the development, we gathered user feedback to refine the platform’s design, ensuring its applicability in clinical settings. This iterative process pinpointed two key usability challenges – independent armband use and application navigation – essential for enhancing the platform’s usability and effectiveness beyond supervised therapy. While these aspects may not significantly impact the feasibility of using the platform in supervised therapy sessions, they could potentially hinder its adoption in unsupervised settings, such as at home.

To enhance the usability and user experience of the MyoGuide platform for future application, we are considering redesigning the Myo armband to address challenges such as independent donning and doffing. Potential adjustments may include increased adjustability in size or implementing a buckle design.

With regard to independent MyoGuide use, both the SUS and the survey on future home use revealed some hesitancy around using MyoGuide without the assistance of a therapist. Recognizing the importance of addressing these usability issues, we are actively working on a revised version of the application for the future study. The updated version aims to minimize the need for button pressing, offer clearer instructions, and enhance navigational simplicity. These improvements are specifically intended to accommodate stroke patients with limited prior experience in mobile technology, fostering a user-friendly interface tailored to their needs and technological comfort levels.

Addressing these issues is crucial for promoting the effectiveness and acceptability of the platform outside of supervised therapy sessions in the future. It is important to note that this study focused on the feasibility and usability of the MyoGuide platform in a supervised setting, with clinical effectiveness remaining a focal point for future research.

## Conclusions

This study introduces the MyoGuide training platform and demonstrates usability in a clinical setting for stroke rehabilitation, with the assistance of a therapist. The findings highlight the potential of MyoGuide for wrist extension training in patients across a wide range of impairment levels. However, certain usability challenges, such as donning/doffing the armband and navigating the application, need to be addressed to enhance the user experience. However, this study focused on overall usability rather than pinpointing specific components of the application that may need refinement. In future usability tests, we will prioritize addressing navigation challenges and identifying specific components requiring improvement. It’s important to acknowledge the limitation of our study’s low sample size, which may impact the generalizability of our findings. Larger and more diverse participant groups would provide a more robust understanding of MyoGuide’s applicability across a broader range of stroke survivors. Additionally, further improvements are needed in terms of stratification strategies, such as adjusting the starting difficulty based on the level of impairment or providing customizable training programs before the tool can be tested during (i) the acute stage in hospitals or (ii) continued training in minimally supervised settings, e.g., community centers or at the patient’s home.

### Electronic supplementary material

Below is the link to the electronic supplementary material.

Supplementary Material 1

Supplementary Material 2

Supplementary Material 3

Supplementary Material 4

## Data Availability

All data generated or analysed during this study are included in this published article and its supplementary information files.

## Abbreviations

FMA-UE: Fugl-Meyer Assessment for Upper Extremity
SUS: System Usability Scale
ADL: Activities of Daily Living
sEMG: Surface Electromyography
LoD: Level of Difficulty
