# Supplementary material for: A usability study on mobile EMG-guided wrist extension training in subacute stroke patients-MyoGuide
Source: J Neuroeng Rehabil. 2024 Mar 21;21:39. doi: 10.1186/s12984-024-01334-9 (PMC10956308; doi:10.1186/s12984-024-01334-9)
Supplement: Supplementary file 4 — Supplementary Material 4 [file 12984_2024_1334_MOESM4_ESM.docx]

**Therapist 1**

"Distinguished from traditional rehabilitation devices, the portability and ease of use of MyoGuide leaves a strong impression, laying the foundation for its future applications in home rehabilitation and remote medical care. In the course of treatment, my primary task was to assist patients in finding the optimal position for wearing the Armband, guide them to follow the prescribed program, provide reminders when patients use excessive compensatory movements, and instruct on the correct application of force. When patients experience spasticity, appropriate methods were used to help relax the muscles. I was pleasantly surprised to observe that the majority of patients showed a keen interest in this emerging treatment method and quickly grasped the training techniques. Younger patients or those who frequently use mobile devices demonstrated greater initiative during training and increased confidence in independently operating the system. The initial hand function levels varied among patients. For those with better functional levels, MyoGuide helped improve muscle strength and control in a shorter amount of time. Patients with muscle strength at lower levels (approx. 1-2 on MRC rating) benefited from real-time feedback on muscle contractions during training, leading to better perception of the affected upper limb. The precise feedback, combined with the design of completing gaming tasks, effectively motivated patients to engage in more proactive training."

**Therapist 2**

"The training setup provides excellent online feedback, allowing patient to visualize their performance. They can complete their training tasks in a gamified environment, which greatly encouraged them. The setup was simple and easy for patients to understand. The device was compact and lightweight, enabling even early bedridden patients to participate. Although patients may experience fatigue during the game, the setup adjusted the difficulty on a daily basis, enabling most patients to persevere without any discomfort. This specific targeted training, coupled with an adequate amount of training time, enabled patients to see their progress in a short period, boosted their confidence and positively influenced their belief in rehabilitation."
